# Genotyping of the gene polymorphisms *FASL* 844C/T and *FAS* 670A/G in patients with idiopathic infertility in Iraq

**DOI:** 10.1590/1806-9282.20241860

**Published:** 2025-06-16

**Authors:** Abbas Ali Manshd, Mohammed Ali Alaboudi, Abbas Abdulameer Al-Raad

**Affiliations:** 1 Sawa University, College of Health and Medical Technology, Department of Medical Laboratories – Al-Muthanna, Iraq.; 2 Al-Muthanna University, College of Education for Pure Sciences, Department of Biology – Al-Muthanna, Iraq.

**Keywords:** FAS, FASL, Genotype, Male infertility, Polymorphism

## Abstract

**OBJECTIVE:**

The failure to conceive following 12 months of unprotected intercourse is referred to as infertility. About 35% of the instances include women and 30% involve men.

**AIM:**

The aim of this study was to investigate the gene polymorphisms *FASL*-844C/T and *FAS*-670A/G in the promoter region to evaluate susceptibility to male infertility.

**METHODS:**

We have investigated single-nucleotide polymorphisms of *FAS*-670A/G gene and *FASL*-844C/T gene in 100 subjects. A total of 50 male patients with infertility constituted the infertility group and another group consisting of 50 apparently healthy individuals comprised the control group.

**RESULTS:**

The prevalence rate of *FA*S-670A/G gene polymorphism was statistically significant among both azoospermia and oligospermia subgroups, the homozygous mutant genotype GG (p=0.014 and p=0.043), and mutant *allele* G (p=0.001 and p=0.004), respectively. Also, *FASL*-844C/T gene polymorphism was statistically significant among both azoospermia and oligospermia subgroups, the homozygous mutant genotype TT (p=0.032 and p=0.032), and mutant *allele* T (p=0.007 and p=0.002), respectively.

**CONCLUSIONS:**

*FAS* homozygous mutant genotype (GG) and *FASL* homozygous mutant genotype (TT) act as etiologic factors in male infertility.

## INTRODUCTION

The World Health Organization defines infertility as the inability to get pregnant after 12 months of unprotected sexual ­activity^1^. It now affects 10–15% of couples globally. Its incidence has increased over the years^2,3^.

*FAS* and *FASL* are known to participate in apoptosis. *FASL* plays a crucial role in the development of the normal immune system and the maintenance, regulation, and ­function of homeostasis^4^.

The *FAS* and *FASL* genes have been revealed to contain several polymorphism variations. More consideration has been given to these functional polymorphisms because of their functions in controlling gene expression^5^. Functional polymorphisms have been discovered within the *FAS* promoter region and *FASL* genes. These polymorphisms, which are −844C/T in the *FASL* gene and −670A/G in the *FAS* gene, may raise the risk of infertility and cancer^6^. The most prevalent single-­nucleotide polymorphism (SNP) within the *FAS* promoter region is *FAS*-670A/G polymorphism. The STAT1 transcription factor's binding site is altered when a G is substituted for an A at −670 base pairs, which lowers the level of this gene's transcription^7^.

The transcriptional factors can be impacted by the *FASL*-844C/T and *FAS*-670A/G polymorphisms and result in a considerable unusual apoptosis that is linked to the susceptibility of disease occurrence^8^. Recent research on male infertility revealed a substantial correlation between abnormal semen parameters and excessive or not enough germ cell apoptosis in ejaculated sperm^9^.

The current study aimed to investigate the gene polymorphisms *FASL*-844C/T and *FAS*-670A/G in the promoter region to evaluate susceptibility to male infertility.

## METHODS

### Subject

This study was conducted on 50 male infertile patients with age ranging between 20 and 43 years, who attended the Infertility and IVF Center in Al-Sader City Hospital for the period from November 2023 to May 2024. A total of 50 healthy subjects without any history of systemic disease were included as the control group. This study excluded any infertile men with reproductive system disorders, surgical histories, diagnosed varicocele, individuals with cryptorchidism and testicular tumors, or those receiving radiotherapy and chemotherapy. A volume of 2 mL of blood samples was used to extract the DNA, and amplification refractory mutation system-polymerase chain reaction (ARMS-PCR) was used to find polymorphisms in the promoter region of the *FAS*/*FASL* genes, including *FASL*-844C/T and *FAS*-670A/G polymorphisms.

### Genotyping

The Tetra-ARMS-PCR technique was performed for the detection of *FASL* C-844T and *FAS* A-670G gene polymorphisms from patient and healthy control blood samples. The blood samples were used to extract genomic DNA using the gSYNCTM Genomic DNA extraction kit, and the purity and concentration of the DNA were checked using a NanoDrop ­spectrophotometer at 260/280 nm, where it was found to range between 1.7 and 2.1, with an average purity equal to 2 and a mean DNA concentration of 20 ng/μL (10–40 ng/μL). The T-ARMS-PCR master mix was performed for each sample using AccuPower PCR PreMix Kit according to manufacturer's instructions. The results were analyzed by loading the ARMS-PCR products on 2% agarose gel. Table 1 lists the ARMS-PCR primers for the genes *FASL*-844C/T and *FAS*-670A/G.

**Table 1:** Sequence of primers and the size of their products.

Primer	Sequence (5’-3’)		Product size
*FAS*-670A/G gene	FO	"GGG GCT ATG CGA TTT GGC TTA AGT TGTT"	A allele 158 bp G allele 207 bp
	RO	"GTA GTT CAA CCT GGG AAG TTG GGG AGGT"	
	FI	"TTT TTC ATA TGG TTA ACT GTC CAT TCC CGG"	
	RI	"GCA ACA TGA GAG GCT CAC AGA CGG TT"	
*FASL*-844C/T gene	FO	"TGC AGT TAA CTA CGA TAG CAC CAC TGCA"	C allele 192 bp T allele 145 bp
	RO	"AGG AGG AAA ACA TCT GTT GCC ATC ATCT"	
	FI	"GGC AAA CAA TGA AAA TGA AAA CAT GGC"	
	RI	"AAC CCA CAG AGC TGC TTT GTA TTG CA"	

FO: forward outer; RO: reverse outer; FI: forward inner; RI: reverse inner.

### Statistical analysis

Data were either normally distributed or not normally distributed and were recorded in a Microsoft Excel spreadsheet. Statistical analysis was carried out with the SPSS v. 0.26 ­software (chi-square test, independent sample t-test, and Spearman's and Pearson's correlation coefficients) after translating data into codes. A p<0.05 was considered statistically significant.

## RESULTS AND DISCUSSION

### Demographic and clinical parameters

When controls and infertile patients were compared in terms of age, the largest rate of infertility was found among young adult patients, especially those under the age of 30 years. The mean age of the infertile patients and controls were 31.3±11.23 and 32.6±12.11 years, respectively. Age did not significantly differ across the research groups (p=0.133). Family history is a significant contributing factor to infertility. In addition, the present study's findings revealed that the prevalence of oligospermia was higher in patients with a positive family history (68%) than in those with no family history (32%; p=0.05).

This agrees with a study conducted by Attia et al.^10^ who discovered no statistically significant differences in the ages of oligospermic and azoospermic patients in comparison to controls (33±4.8, 37±5.4, and 33±5.3 years, respectively; p>0.24). Also, Ejzenberg et al.^11^ discovered no statistically significant differences between controls and infertile patients in terms of age. Shabani et al.^12^ found that testosterone levels diminish over time as men age, but this loss varies greatly across men and may be influenced by variables, including drugs, obesity, genetics, alcoholism, and chronic illnesses.

In a previous study, 13.5% of the patients that were included had a positive family history of male infertility, suggesting that male infertility may be caused by hereditary factors^13^. Given the genetic foundation of infertility, family history is a portion of the patient's history that is becoming more significant^14^.

### Genotype and *allele* distribution in patient and control groups for *FAS*-670A/G and *FASL*-844C/T gene polymorphisms

Tetra-ARMS-PCR technique was used to identify the *FAS* A-670G gene polymorphism distribution. At this locus, there are three genotypes for *FAS*-670A/G such as AA, AG, and GG with band sizes of 158 pb, 158/207 pb, and 207 pb, respectively. In this study, we genotyped the *FAS*-670A/G gene polymorphism in infertile patients to establish a link between their clinical features and the genotypes/allotypes.

The genotypes’ relative frequency of the *FAS*-670A/G gene polymorphism in the azoospermia subgroup and control group was as follows: AA (28 and 54%), AG (24 and 26%), and GG (48 and 20%), respectively, as shown in Table 2.

**Table 2:** Comparison between azoospermia subgroup and control group by genotype.

Genotype/*allele*	Azoospermia subgroup (n=25)			Control group (n=50)			p-value	Odds ratio	95%CI	Etiologic fraction	Preventive fraction
	No.	Frequency	%	No.	Frequency	%					
*AA*	7	0.28	28	27	0.54	54	0.036	0.331	0.117–0.932	***	0.292
*AG*	6	0.24	24	13	0.26	26	0.851	0.898	0.294–2.739	***	0.034
*GG*	12	0.48	48	10	0.2	20	0.014	3.692	1.296–10.517	0.397	***
*A*	20	0.4	40	67	0.67	67	0.001	0.328	0.162–0.663	***	0.318
*G*	30	0.6	60	33	0.33	33	0.001	3.045	1.508–6.149	0.973	***

CI: confidence interval.

*** Etiologic fraction for genotype AA is the source (reference), its value must be 1.

The corresponding frequency in the oligospermia subgroup and the control group was as follows: AA (32 and 58%), AG (28 and 24%), and GG (40 and 18%), respectively, as shown in Table 3.

**Table 3:** Comparison between oligospermia subgroup and control group by genotype.

Genotype/*allele*	Oligospermia subgroup (n=25)			Control group (n=50)			p-value	Odds ratio	95%CI	Etiologic fraction	Preventive fraction
	No.	Frequency	%	No.	Frequency	%					
*AA*	8	0.32	32	29	0.58	58	0.036	0.34	0.124–0.936	***	0.294
*AG*	7	0.28	28	12	0.24	24	0.707	1.231	0.414–3.655	0.069	***
*GG*	10	0.4	40	9	0.18	18	0.043	3.037	1.034–8.920	0.352	***
*A*	23	0.46	46	70	0.7	70	0.004	0.365	0.181–0.736	***	0.299
*G*	27	0.54	54	30	0.3	30	0.004	2.739	1.358–5.524	0.3	***

CI: confidence interval.

*** Etiologic fraction for genotype AA is the source (reference), its value must be 1.

The distribution of the *FASL*-844C/T gene polymorphism was detected by the Tetra-ARMS-PCR technique. At this locus, there are three genotypes for *FASL*-844C/T such as CC, CT, and TT with band sizes of 192 pb, 192/145 pb, and 145 pb, respectively. In this study, we genotyped the *FASL*-844C/T gene polymorphism in infertility patients to determine the relationship between their clinical features and the genotypes/allotypes.

The genotypes’ relative frequency of the *FASL*-844C/T gene polymorphism in the azoospermia subgroup and control group was as follows: CC (32 and 54%), CT (24 and 26%), and TT (44 and 20%), respectively, while the corresponding frequency in the oligospermia subgroup and control group was as follows: CC (28 and 56%), CT (28 and 24%), and TT (44 and 20%), respectively, as shown in Table 4.

**Table 4:** Comparison between azoospermia and oligospermia subgroups and control group by genotype.

Genotype/*allele*	Azoospermia subgroup (n=25)			Control group (n=50)			p-value	Odds ratio	95%CI	Etiologic fraction	Preventive fraction
	No.	Frequency	%	No.	Frequency	%					
*CC*	8	0.32	32	27	0.54	54	0.075	0.4	0.146–1.098	***	0.235
*CT*	6	0.24	24	13	0.26	26	0.851	0.898	0.294–2.739	***	0.034
*TT*	11	0.44	44	10	0.2	20	0.032	3.142	1.099–8.986	0.356	***
*C*	22	0.44	44	67	0.67	67	0.007	0.387	0.192–0.776	***	0.28
*T*	28	0.56	56	33	0.33	33	0.007	2.584	1.287–5.187	0.281	***
**Oligospermia subgroup**											
*CC*	7	0.28	28	28	0.56	56	0.024	0.305	0.108–0.861	***	0.313
*CT*	7	0.28	28	12	0.24	24	0.707	1.231	0.414–3.655	0.069	***
*TT*	11	0.44	44	10	0.2	20	0.032	3.142	1.099–8.986	0.356	***
*C*	21	0.42	42	68	0.68	68	0.002	0.34	0.169–0.687	***	0.313
*T*	29	0.58	58	32	0.32	32	0.002	2.934	1.455–5.917	0.313	***

CI: confidence interval.

*** Etiologic fraction for genotype CC is the source (reference), its value must be 1.

The current study revealed that there was a considerable difference in the control group compared to the patient group when both groups were compared with azoospermia and oligospermia subgroups, with p<0.05 for genotype and allele frequencies. Statistical analysis showed that *FASL*-844C/T and *FAS*-670A/G were risk factors for infertility, and there was an association between their polymorphism and infertility.

Our findings supported those of research done by Hassan et al.^15^, who showed that the proportions of the GA and AA genotypes were statistically significantly greater in the group of patients with severe oligozoospermia (p<0.01) than in the group of patients with normozoospermia (30 vs. 20% and 60 vs. 0%, respectively).

The current findings were in line with those of research done by Balkan et al.^5^, who showed a correlation between the high propensity for idiopathic azoospermia and the homozygous genotypes GG and AA in the *FAS*-670A/G gene polymorphism. Furthermore, it was shown that the heterozygous AG genotype was considerably lower in patients compared to controls, suggesting that heterozygous genotypes in this ­variation may have had a protective effect against idiopathic azoospermia. The *FAS*-670 A/G gene polymorphism is in fact supported by these findings as a risk factor for male infertility in the Turkish community.

Ji et al.^16^ revealed a strong correlation between sperm apoptosis and semen quality and functional polymorphisms in the *FAS*-670G/A gene promoter (OR 0.48; 95%CI 0.28–0.83). When compared to individuals with the AA genotype, those with the *FAS*-670 GG genotype exhibited lower rates of apoptosis, which were associated with lower sperm concentration and poorer sperm motility (mean: 90.15×10^6^/mL; 72.44×10^6^/mL; and 58.14×10^6^/mL, respectively, p=0.001).

Asgari et al.^8^ discovered that the presence of the TT, CT, and CT+TT genotypes was related to an elevated risk of infertility. The TT genotype was also associated with a 2.02-fold higher risk of infertility that was statistically significant (p=0.03). Furthermore, the −844T *allele* was more common in infertile males (59.8%) compared to controls (50.9%). As a result, this difference might be regarded as a risk factor for male infertility in the Western Iranian community.

Idiopathic azoospermia has been discovered to be highly associated with the polymorphism in *FASL*-844C/T. In other words, Han Chinese men may be predisposed to severe oligozoospermia or idiopathic azoospermia due to a familial predisposition^9^.

## CONCLUSION

*FAS* homozygous mutant genotype (GG) and *FASL* homozygous mutant genotype (TT) acted as risk factors for male infertility.
